# Guest Edited Collection: Nanotechnology in agriculture

**DOI:** 10.1038/s41598-020-73198-7

**Published:** 2020-09-25

**Authors:** Alejandro Pérez-de-Luque

**Affiliations:** IFAPA, Centro Alameda del Obispo, Área de Mejora y Biotecnología, Avda. Menédez Pidal s/n, Apdo. 3092, 14004 Córdoba, Spain

**Keywords:** Plant sciences, Nanoscience and technology

## Abstract

Agriculture must overcome several challenges in order to increase—or even maintain—production, while also reducing negative environmental impact. Nanotechnology, fundamentally through the development of smart delivery systems and nanocarriers, can contribute to engineering more efficient and less contaminant agrochemicals. This Collection presents recent related works, covering nanodevices that improve crop protection against pests and diseases, nanoformulations for enhancing plant nutrition, and nanomaterials strengthening the general crop performance.

The United Nations Food and Agriculture Organization estimates that the world population will approach 10 billion people by 2050. This poses a major challenge for agriculture, as in order to achieve the required production increases, scarce land and water resources must be overcome, together with the negative impacts of climate change^1^. In addition, farming efficiency relies on the massive use of agrochemicals for maintaining crop yields, which pushes against the sustainability of the system. Research aiming to minimize the negative impact of agrochemicals has been a priority for a long time, and nanotechnology provides a promising new tool, for both fertilization^2^ and crop protection^3^.

Nanodevices can benefit agriculture through the development of more efficient and less contaminant agrochemicals, using nanocarriers and smart delivery systems for controlled release. These nanoformulations provide several advantages, such as protection for the active substances, increased solubilization, and facilitating penetration and internalization into plant and target organism tissues^2–4^. All these should lead to an increased effectiveness of the agrochemicals, reducing waste, dosage, and—as a consequence—minimizing any adverse effects on the environment and non-target organisms.

Nevertheless, research must be done to help specify the nanomaterials that are to be used. As massive applications are expected in the fields for farming, such materials should be environmentally safe and biologically compatible, in addition to providing a cheap and industrially scalable synthesis^4^.

Finally, in order for all of this research to reach practical applications, field experimentation and testing are necessary. Field experiments for agriculture are like clinical trials for medicine: it is the only way to validate promising results obtained under laboratory and controlled conditions. Despite the fact that most work to date has been developed under laboratory settings, new results involving field trials and realistic conditions are now being published^5–8^, showing the real potential of these nanotechnologies for agriculture.

## Collection overview

At the time of this Collection’s launch, a high proportion of the work falls under the section ‘Nanodevices for crop protection’. This research relates to the use of nanoformulations for fighting against biotic and abiotic stresses in plants, with methods that directly affect insect pests, through disruption of their immune and reproductive system^9^ or acting as attractants^10^. Some others show the antimicrobial activity of nanoparticles^8,10–12^, present nanosorbents for improving and increasing the properties of plant allelochemicals^13^, and stimulate the plant natural defences, using nanomaterials^14^ or through loading nanocarriers with resistance inducers^15^.

Some works have focused on ‘Nanodevices for crop nutrition’, for example, as sources of phosphorus and iron using an industrially scalable protocol^16^. Two other articles deal with nanohydroxyapatite as a viable carrier of plant nutrients. One checks its negligible impact on the rhizosphere microbial community, and reports a lack of effect as a source of P for soybean^17^, whereas the other explores the behaviour of this compound, and how its morphology at the nanoscale determines release of phosphate and nitrate ions^18^.

There are also ‘Other applications and effects’ that have been tested and developed, such as efficient delivery of plant hormones and growth regulators which positively affect crop development^6,19^, or active molecules that are able to modulate stress responses^5^. In addition, there are reports about how priming seeds with nanomaterials leads to improved growth and yield in some crops^7,20^. Finally, a method for removing toxic chromium from irrigation water using iron nanoparticles^21^ is included, showing a way for recovering contaminated water sources for farming.

Some works are currently under review and there is still room for many more in the Collection. As stated above, field trials are a must, and aiming to solve real agricultural problems should drive future research, but without forgetting basic research, which is the source for practical applications. To paraphrase Richard Feynman, in agriculture “there’s plenty of room at the bottom”.
